# Modeling vaccination and control strategies for outbreaks of monkeypox at gatherings

**DOI:** 10.3389/fpubh.2022.1026489

**Published:** 2022-11-25

**Authors:** Pei Yuan, Yi Tan, Liu Yang, Elena Aruffo, Nicholas H. Ogden, Jacques Bélair, Julien Arino, Jane Heffernan, James Watmough, Hélène Carabin, Huaiping Zhu

**Affiliations:** ^1^Laboratory of Mathematical Parallel Systems (LAMPS), Department of Mathematics and Statistics, York University, Toronto, ON, Canada; ^2^Canadian Centre for Diseases Modeling (CCDM), York University, Toronto, ON, Canada; ^3^School of Mathematics and Statistics, Northeast Normal University, Changchun, China; ^4^Public Health Risk Sciences Division, National Microbiology Laboratory, Public Health Agency of Canada, Saint-Hyacinthe, QC, Canada; ^5^Département de Mathématiques et de Statistique, Université de Montréal, Montréal, QC, Canada; ^6^Department of Mathematics, University of Manitoba, Winnipeg, MB, Canada; ^7^Department of Mathematics and Statistics, York University, Toronto, ON, Canada; ^8^Department of Mathematics and Statistics, University of New Brunswick, Fredericton, NB, Canada; ^9^Département de Pathologie et Microbiologie, Faculté de médecine vétérinaire, Université de Montréal, Saint-Hyacinthe, QC, Canada; ^10^Département de médecine sociale et préventive, École de santé publique de l'Université de Montréal, Montréal, QC, Canada; ^11^Centre de Recherche en Santé Publique (CReSP) de l'université de Montréal et du CIUSS du Centre Sud de Montréal, Montréal, QC, Canada; ^12^Groupe de Recherche en Épidémiologie des Zoonoses et Santé Publique (GREZOSP), Université de Montréal, Saint-Hyacinthe, QC, Canada

**Keywords:** monkeypox, vaccination strategy, ring vaccination, gatherings, testing, modeling, control

## Abstract

**Background:**

The monkeypox outbreak in non-endemic countries in recent months has led the World Health Organization (WHO) to declare a public health emergency of international concern (PHEIC). It is thought that festivals, parties, and other gatherings may have contributed to the outbreak.

**Methods:**

We considered a hypothetical metropolitan city and modeled the transmission of the monkeypox virus in humans in a high-risk group (HRG) and a low-risk group (LRG) using a Susceptible-Exposed-Infectious-Recovered (SEIR) model and incorporated gathering events. Model simulations assessed how the vaccination strategies combined with other public health measures can contribute to mitigating or halting outbreaks from mass gathering events.

**Results:**

The risk of a monkeypox outbreak was high when mass gathering events occurred in the absence of public health control measures. However, the outbreaks were controlled by isolating cases and vaccinating their close contacts. Furthermore, contact tracing, vaccinating, and isolating close contacts, if they can be implemented, were more effective for the containment of monkeypox transmission during summer gatherings than a broad vaccination campaign among HRG, when accounting for the low vaccination coverage in the overall population, and the time needed for the development of the immune responses. Reducing the number of attendees and effective contacts during the gathering could also prevent a burgeoning outbreak, as could restricting attendance through vaccination requirements.

**Conclusion:**

Monkeypox outbreaks following mass gatherings can be made less likely with some restrictions on either the number and density of attendees in the gathering or vaccination requirements. The ring vaccination strategy inoculating close contacts of confirmed cases may not be enough to prevent potential outbreaks; however, mass gatherings can be rendered less risky if that strategy is combined with public health measures, including identifying and isolating cases and contact tracing. Compliance with the community and promotion of awareness are also indispensable to containing the outbreak.

## Introduction

Monkeypox, a zoonosis, has been recorded since early May 2022 in at least 30 non-endemic countries including Spain, the United States, Germany, the United Kingdom, France, and Canada (1). As of 21 July 2022, the cumulative number of confirmed cases exceeded 15,000 globally (2). On 23 July 2022, the World Health Organization (WHO) declared monkeypox a Public Health Emergency of International Concern (PHEIC) due to outbreaks in multiple countries and continents (3). The Public Health Agency of Canada (PHAC) reported 1,410 cases of monkeypox as of 14 October 2022, mostly occurring in Quebec, Ontario, and British Columbia (4). The unusual outbreak emerged in non-endemic areas of the world associated with transmission among gay, bisexual, and other men who have sex with men (gbMSM) (5). Although at the time of writing this paper, the epidemic was declining, there remains a pressing need to understand the epidemic and potential control methods (5–7).

Monkeypox virus, which is closely related to smallpox, is an enveloped double-stranded DNA virus, with two clades, the Central African clade and the West African clade (7, 8). The former is more virulent with reported fatality rates in Africa of 10% for the Central Africa clade and 3.6% for the West African clade (7). The incubation period ranges from 5 to 21 days, after which infected individuals may initially have flu-like symptoms, then, 1–3 days later, a characteristic skin rash develops. The recovery period may take 2–4 weeks (8). In the recent outbreak, there are atypical clinical observations. The majority of the patients are gbMSM who reported genital lesions which subsequently develop into skin lesions on other body sites, although with more limited distribution than reported in the previous outbreaks (9).

In Canada, control is based on vaccines and non-pharmaceutical interventions including recommendations for testing and isolation of cases, and, where possible, tracing of contacts (10). In June 2022, the National Advisory Committee on Immunization (NACI) released a guideline on using an orthopoxvirus (Imvamune^®^) vaccine with potential efficacy against monkeypox (11). The guideline recommends pre-exposure prophylaxis (PrEP) vaccination for adults at high risk of exposure (occupational or otherwise) and also post-exposure prophylaxis (PEP).

After 2 years of restrictions on gatherings due to the control and prevention of the COVID-19 pandemic, mass gatherings related to festivals and ceremonies are now allowed with no attendance limitations (12). In many Canadian provinces, local festivals recorded attendance close to the pre-pandemic level (12–15), which has led to concerns about the spread and possible outbreaks of monkeypox. WHO also expressed concerns that more infections could arise in Europe and elsewhere (16) due to private and social gatherings during festivals, parties, and holidays. In fact, in the United States, many cases were reported linked to large social gatherings, such as pride events, pool parties, and bathhouses (17, 18). Consequently, it is essential to assess the effect of gathering events on monkeypox transmission to inform public health on the most effective control measures.

Transmission risk at a gathering is mainly associated with the gathering size (19) and is proportional to the population density at the gathering place (20). Using an individual-based model, Moritz et al. (21) showed that for a mass gathering event (MGE) with 200,000 participants, there is a 23.6% increase in positive cases attributed to MGE for the transmission of COVID-19. Also, the effect of increased density of contacts during Hajj was estimated to generate a 78-fold increase in meningococcal infection that impacts not only pilgrims but also the local population (22).

To investigate the dynamics of monkeypox and provide information to public health for prevention and control, especially at gatherings, we established a SEIR modeling framework to assess the effect of the vaccination and other control methods. The vaccination in a high-risk group and ring vaccination strategy along with testing and isolation of cases and contact tracing, as well as the possible interventions during gathering events, are also considered. We mainly focused on assessing the effectiveness of public health control measures, including preventive vaccination or vaccination post-exposure, to simulate the scenarios of gathering with different numbers of attendees and different levels of interventions to inform public health decision-making. Our findings suggested that reactive ring vaccination may itself not be enough; however, if close contacts of cases can be identified, vaccinated, and isolated, an outbreak after MGEs may be prevented.

## Methods

### Modeling overview

The vast majority of reported cases occurring in the recent outbreaks in non-endemic areas have been linked to specific high-risk locations and populations. Hence, to better capture the infection dynamics within different risk settings, we considered the population to be divided into two subgroups: a low-risk population (LRG), which is defined as individuals who behave in such a way that their possibility of becoming infected is reduced, and a high-risk population (HRG), which is defined as individuals whose behavior makes them at higher risk of acquiring the infection. For simplicity, henceforth, we use subscript 1 for the LRG and subscript 2 for the HRG. Those two groups interact between and within groups as represented by a contact matrix (*c*_*ij*_, *i, j* = 1, 2), defined using the assumptions in Yuan et al. (23). We assumed that there is no movement of population between the risk groups unless there is a gathering event such that a proportion of LRG people may become part of the HRG.

**Figure 1:**
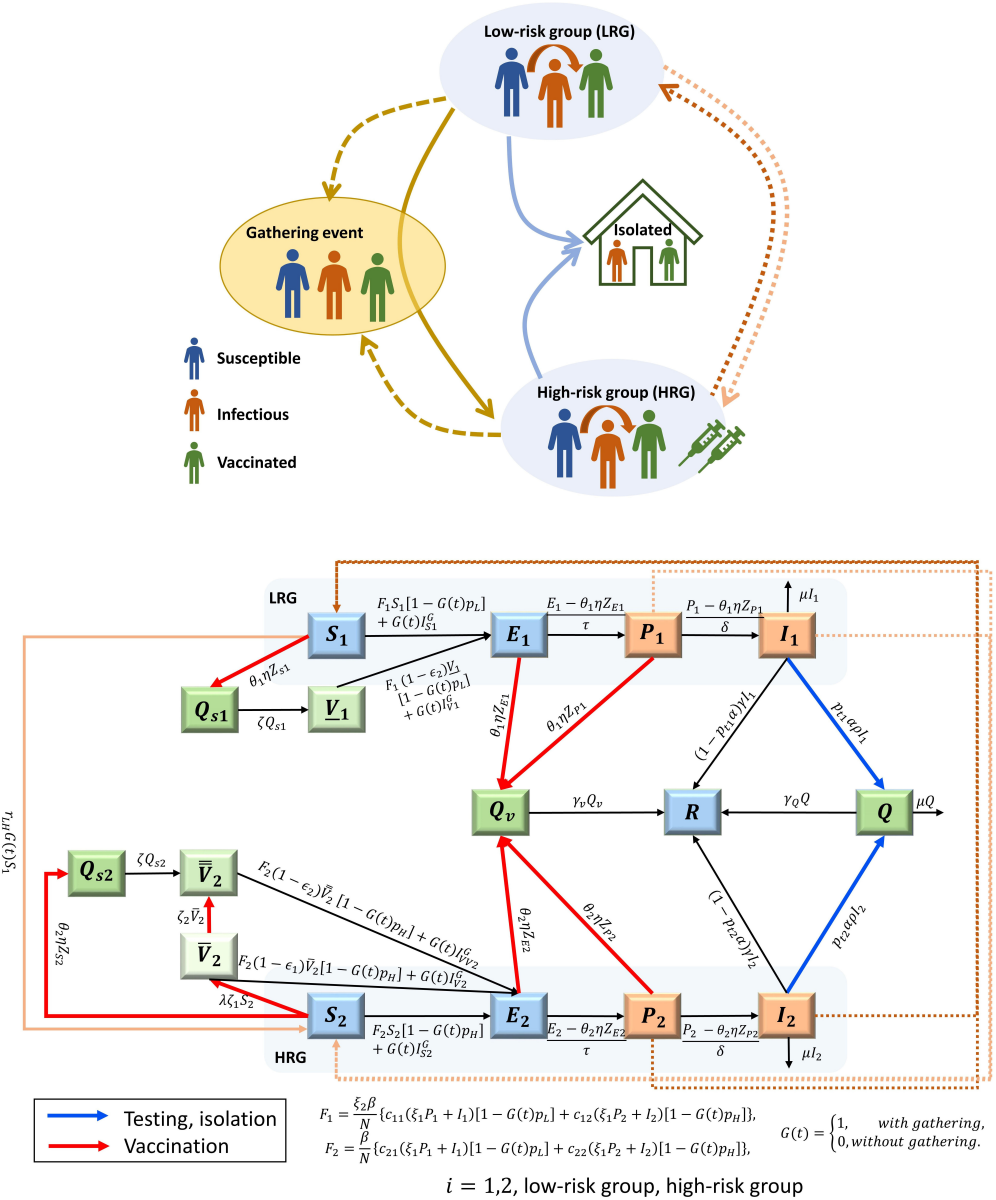
Schematic diagram **(A)** and flow chart **(B)** of the MPX transmission among the population classified with low-risk group (LRG, ***i*** = 1) and high-risk group (HRG, ***i*** = 2), considering the gathering event. In groups i: Susceptible (S_i_), Exposed (E_i_), infectious, prodromal phase (P_i_), infectious, acute phase, with rash symptom (I_i_), Recovered R; Quarantined (Q), Quarantined and vaccinated (Q_v_); Quarantined and Susceptible (Q_si_); partially vaccinated HRG individuals, ${\bar{\text{V}}}_{2}$, fully vaccinated HRG individuals ${\bar{\bar{\text{V}}}}_{2}$, fully vaccinated LRG individuals (born before 1972), *V*_1_. The individuals in the orange compartments are infectious, while those in dark green compartments are quarantined and hence not involved in the transmission. The compartments of partially and fully vaccinated are presented with light and middle green colors respectively. The arrow with red color represents the process of vaccinating the HRG individuals and the exposures from contact tracing, while the arrow with blue color represents the testing and isolation of symptomatic infections. The dash lines show the transmission routes between the LRG and HRG. The description of the parameters shown in the flow diagram can be found in Tables 3, 4.

The infection dynamic follows the SEIR framework, which is extended to include the prodromal stage, vaccinated (partially, fully), and quarantined (tested and confirmed, vaccinated, and susceptible). Susceptible (S) individuals become infected, and move to the exposed compartment (E), after encountering an infectious individual from either LRG or HRG, assuming that the latter group is with higher susceptibility than the first group. After a latent period, the prodromal stage (P) begins, and during this phase no symptoms are apparent, but the individual can shed the virus (24). This period is then followed by the symptomatic infectious stage (I) and then recovery (R) occurs. Infectious individuals with symptoms may be tested, then quarantined (Q), while their contacts, which might be susceptible, exposed, or pre-symptomatic, can be vaccinated and quarantined (Q_s_ or Q_v_) to prevent any further spread of the infection. Although the model does not include demographics, we assumed that infection-related death might occur among infectious individuals. Isolated individuals who develop infection will remain isolated until recovery. Individuals in both prodromal and symptomatic infectious stages can transmit the infection; however, infections in the prodromal phase are assumed to be less infectious than those in the symptomatic phase. The population structure and flow diagram of the disease are shown in Figure 1.

We included the current vaccination process in Canada (11) to explore its effectiveness by assuming that only high-risk people and close contacts of confirmed cases will be vaccinated. We also examined the public health measures of testing and isolating cases. Model assumptions, variables, and parameters are summarized in Tables 1–5, and the model equations are presented in Supplementary material S1.

**Table 1:** Model assumptions.

**Model assumptions**	
Population classification	• The human population is divided into two groups, low-risk group (LRG, i = 1) and high-risk group (HRG, i = 2).
Demographic	• Immigration, birth, and natural death of the population are ignored. The mortality from infection is considered.
Monkeypox transmission	• Infectious individuals in both prodromal phase (with lower infectiousness ξ_1_, assumed to be 75% of that during the rash stage) and acute phase can transmit the virus. • The tested positive, confirmed and isolated individuals and the vaccinated and quarantined individuals are not involved in the transmission. • The susceptibility of LRG individuals is lower than that of HRG individuals, with a scaling factor ξ_2_ (assumed to be 0.05). • The contacts among LRG individuals mainly come from the LRG individuals, with the proportion *k*_1_ (assumed to be 0.6) of the contact rate baseline in the human population. • The contact rate in the HRG is higher than that in the LRG, by a factor *k*_2_ of the contact rate baseline (assumed to be 1.3). • The contact between HRG individuals and LRG individuals is low, accounting for (1−*k*_1_) of the contact rate baseline. • The vertical transmission is ignored.
Testing and isolation	• Individuals with rash symptoms will go to hospital seeking medical help and then be tested, confirmed and isolated and this process may take 1/ρ days. • Proportion of confirmed infections (α) will comply with the isolation strategy.
Vaccination	• The two-dose vaccine considered is Imvamune^®^, and a second dose is offered 28 days after the first dose. • Individuals receiving the first dose after 2 weeks when the immune response is detectable, and the second dose of Imvamune after 6 weeks when the immune response peaked, are considered as partially vaccinated and fully vaccinated, respectively. Given the vaccination campaign against smallpox, individuals born before 1972 are considered to be fully vaccinated. • Post-Exposure Prophylaxis with contact tracing (PEPCT): Individuals (without symptom) with high-risk exposures to a confirmed case of monkeypox may be vaccinated and then quarantined. • Pre-Exposure Prophylaxis in HRG (PrEPH): Susceptible individuals in HRG may be vaccinated. • The effectiveness of the first and second dose is assumed to be ϵ_1_ and ϵ_2_, respectively.
Gathering event	• During the period of gathering event, the contact rate between LRG and HRG and within HRG may increase κ_1_ and κ_2_ times, respectively. • Some susceptible individuals in the LRG may transit to the HRG.

### Transmission

We used the assumptions of Yuan et al. (23) on the transmission of the monkeypox virus. The probability of transmission per contact was assumed to vary between 12.2 and 24.5% among the HRG and between 0.37 and 0.74%, among LRG, as calculated from the basic reproduction number *R*_0_ derived from our simplified model without public health control measures (see Supplementary material S2 for details) and all the other parameters being fixed (**Tables 3**, **4**).

### Public health interventions

On 10 June 2022, NACI issued interim guidance on the use of Imvamune^®^ in the context of monkeypox outbreaks in Canada (11). Imvamune, initially developed for the prevention of smallpox, is a two-dose vaccine with the second dose administered 28 days after the first one. The immune response is detectable by week 2 after the first dose and peaked at week 6 after dose 2 in a randomized, open-label trial designed to compare the effectiveness of Imvamune with the second-generation replicating smallpox vaccine (11). Given the recent emergence of cases and the use of vaccines, there are no available data indicating the effectiveness of Imvamune vaccination against monkeypox infection; however, studies of vaccine effectiveness (VE) of smallpox vaccine may provide a general estimation. In the context of PEP, the median effectiveness in preventing smallpox disease with vaccination at 1–3 days after exposure was estimated at 80% (30). There are a lot of uncertainties about the effectiveness of Imvamune against monkeypox infection, although some observational studies suggested an efficacy of about 85% among fully vaccinated individuals (11). Hence, in our model, we assumed that the efficacy of the first and second doses ranges between 40 and 60%, and 70 and 85%, respectively.

For simplification, given the extensive vaccine campaign against smallpox until 1972, we assumed that all individuals born before that year are fully vaccinated and therefore protected in the model. We defined here that partially and fully vaccinated represent the individuals who received one or two doses of vaccine, respectively.

#### PEP with contact tracing (PEPCT)

Post-Exposure Prophylaxis with Contact Tracing (PEPCT) in our model means that individuals (without symptoms) with exposure to any confirmed case of monkeypox may be traced, vaccinated, and then quarantined. The effect of the vaccine in PEPCT is not significant as the individuals with exposure will be required to isolate and quarantine until fully recovered.

The proportion of PEPCT is represented by θ_*i*_, (*i* = 1, 2), which should be the product of the vaccination proportion of the traced close contact and the proportion of contacts that could be traced. Also, it takes time to trace and isolate which in fact can be modeled by the parameter 1/η, the average time from exposure to becoming vaccinated and isolated.

We denote by *M*_*I*_1__(*t*) and *M*_*I*_2__(*t*) the number of new daily confirmed monkeypox cases in the LRG and HRG at time *t*, respectively, and are defined as follows:


$$\begin{array}{l} M_{I_{1}}\left(t\right)=\rho I_{1}\left(t\right)\\ M_{I_{2}}\left(t\right)=\rho I_{2}\left(t\right) \end{array}$$

where 1/ρ is the average number of days infectious individuals spend between showing rash symptoms and being tested and confirmed.

Following Yuan et al. (23), the number of close contacts of newly confirmed cases in the LRG, who are in the exposed state *Z*_*E*1_(*t*) and the prodromal phase *Z*_*P*1_(*t*) at time *t* can be calculated as


$$\begin{array}{l} Z_{E1}\left(t\right)=[\xi_{2}\beta \sum_{i=1}^{2}c_{2i}M_{I_{1}}\left(t\right)\frac{c_{11}}{c_{11}+c_{12}}\\ \;+\beta \sum_{i=1}^{2}c_{2i}M_{I_{2}}\left(t\right)\frac{c_{21}}{c_{21}+c_{22}}]\frac{\tau }{\tau +\;\delta }, \end{array}$$

$$\begin{array}{l} Z_{P1}\left(t\right)=[\xi_{2}\beta \sum_{i=1}^{2}c_{2i}M_{I_{1}}\left(t\right)\frac{c_{11}}{c_{11}+c_{12}}\\ \;+\beta \sum_{i=1}^{2}c_{2i}M_{I_{2}}\left(t\right)\frac{c_{21}}{c_{21}+c_{22}}]\frac{\delta }{\tau +\;\delta }. \end{array}$$

Similarly, we obtained the number of close contacts of newly confirmed cases in the HRG, who are in the exposed state *Z*_*E*2_(*t*) and the prodromal phase *Z*_*P*2_(*t*), as


$$\begin{array}{l} Z_{E2}\left(t\right)=[\xi_{2}\beta \sum_{i=1}^{2}c_{2i}M_{I_{1}}\left(t\right)\frac{c_{12}}{c_{11}+c_{12}}\\ \;+\beta \sum_{i=1}^{2}c_{2i}M_{I_{2}}\left(t\right)\frac{c_{22}}{c_{21}+c_{22}}]\frac{\tau }{\tau +\;\delta }, \end{array}$$

$$\begin{array}{l} Z_{P2}\left(t\right)=[\xi_{2}\beta \sum_{i=1}^{2}c_{2i}M_{I_{1}}\left(t\right)\frac{c_{12}}{c_{11}+c_{12}}\\ \;+\beta \sum_{i=1}^{2}c_{2i}M_{I_{2}}\left(t\right)\frac{c_{22}}{c_{21}+c_{22}}]\frac{\delta }{\tau +\;\delta }. \end{array}$$

Also, the number of close contacts of newly confirmed cases that are susceptible in the LRG and HRG is


$$\begin{array}{l} Z_{S1}\left(t\right)=\left(1-\xi_{2}\beta \right)\overset{2}{\underset{i=1}{\sum }}c_{2i}M_{I_{1}}\left(t\right)\frac{c_{11}}{c_{11}+c_{12}}\\ \;+\left(1-\beta \right)\overset{2}{\underset{i=1}{\sum }}c_{2i}M_{I_{2}}\left(t\right)\frac{c_{21}}{c_{21}+c_{22}}, \end{array}$$

$$\begin{array}{l} Z_{S2}\left(t\right)=\left(1-\xi_{2}\beta \right)\overset{2}{\underset{i=1}{\sum }}c_{2i}M_{I_{1}}\left(t\right)\frac{c_{12}}{c_{11}+c_{12}}\\ \;+\left(1-\beta \right)\overset{2}{\underset{i=1}{\sum }}c_{2i}M_{I_{2}}\left(t\right)\frac{c_{22}}{c_{21}+c_{22}}. \end{array}$$

#### PrEPH in HRG (PrEPH)

Pre-exposure prophylaxis in HRG (PrEPH) refers to administering the vaccine to individuals at high risk of exposure to the virus. Hence, in our model, vaccination is only administered to those in HRG. Since people born before 1972 have been vaccinated with the smallpox vaccine, therefore, in the initial state in the model, a proportion of the LRG and HRG populations is considered fully vaccinated for simplicity.

#### Testing and isolation

Testing and isolation are crucial steps to detect monkeypox infections and stop the virus from spreading. Individuals with clinical illnesses where monkeypox is suspected should be tested and proceeded for self-isolation before the negative test result is received, while individuals with positive test results should isolate at home until they recover. However, it can take several days from when infected individuals develop symptoms to seek medical help and then get tested, with large variations depending on the individuals' behaviors. In addition, the recovery period takes 2–4 weeks, and with respect to the isolation strategy may not be total, hence compliance with the isolation strategy for those tested and confirmed monkeypox cases are also included in our model.

### Gatherings

Gathering events, here refer to mass gatherings, are defined by the WHO as “more than a specified number of persons at a specific location for a specific purpose for a defined period of time” (31). Gatherings may contribute significantly to the spread of infectious diseases, as was extensively studied during the SARS-CoV-2 pandemic (19–21).

#### Modeling of gathering event in the absence of a specific intervention

Not only will individuals from HRG attend the gathering event, but also individuals in the LRG will join and some of them may transit to the HRG; therefore, facilitating virus spreading and posing the risk of a possible outbreak. In our model, we denote *G*(*t*) as an indicator parameter if there is a gathering event.


$$G\left(t\right)=\;\left\{ \begin{array}{l} 1,\text{ with gathering event},\\ 0,\text{ without gathering event}. \end{array}\right.$$

The daily transition rate from the low-risk susceptible individuals to the high-risk ones is *r*_*LH*_ days^−1^. During the period of gathering events, we assume that the total number of attendees is *N*_*G*_ and the proportion of attendees from LRG and HRG is *p*_*GL*_ and *p*_*GH*_, respectively. The proportion of LRG and HRG individuals attending the gathering event on day *t* is calculated as


$$\begin{array}{l} p_{L}\left(t\right)=\frac{p_{GL}N_{G}}{N_{1}\left(t\right)},\;p_{H}\left(t\right)=\frac{p_{GH}N_{G}}{N_{2}\left(t\right)}, \end{array}$$

respectively, and the number of infectious attendees during the gathering on day *t* is given by


$$\begin{array}{l} N_{GI}\left(t\right)=p_{L}\left(t\right)\left[P_{1}\left(t\right)+I_{1}\left(t\right)\right]+p_{H}\left(t\right)\left[P_{2}\left(t\right)+I_{2}\left(t\right)\right], \end{array}$$

where *N*_1_(*t*) = *S*_1_(*t*) + *E*_1_(*t*) + *P*_1_(*t*) + *I*_1_(*t*) + *V*_1_(*t*) and $N_{2}\left(t\right)=S_{2}\left(t\right)+E_{2}\left(t\right)+P_{2}\left(t\right)+I_{2}\left(t\right)+{\bar{V}}_{2}\left(t\right)+{\bar{\bar{V}}}_{2}\left(t\right)$. Note that the number of recovered individuals is small and not included for simplicity.

Following Champredon et al. (19), we calculated the expected minimum probability of transmissions per attendee that will occur during the gathering, considering the vaccination of attendees, yielding


$$\begin{array}{l} r_{G}\left(t\right)=1-\left({\left(1-\frac{N_{GI}\left(t\right)}{N_{G}-1}\;\beta \right)}^{c_{G}}\right)\; \end{array}$$

where *c*_*G*_ is the number of effective contacts with an infectious individual during the gathering. An effective contact is defined as a contact where there is physical contact and enough exposure time between individuals which may result in the transmission of monkeypox. Hence, the number of LRG-susceptible individuals infected during the gathering is,


$$\begin{array}{l} I_{S1}^{G}=p_{L}S_{1}r_{G}, \end{array}$$

and the number of fully vaccinated LRG individuals infected during the gathering is,


$$I_{V1}^{G}=p_{L}{\underline{V}}_{1}r_{G}(1-\epsilon_{2}).$$

Similarly, we obtained the number of susceptible, partially vaccinated, and fully vaccinated individuals from HRG infected during the gathering as,


$$\begin{array}{l} I_{S2}^{G}=p_{H}S_{2}r_{G},I_{V2}^{G}=p_{H}{\bar{V}}_{2}r_{G}\left(1-\epsilon_{1}\right),I_{VV2}^{G}=p_{H}{\bar{\bar{V}}}_{2}r_{G}\left(1-\epsilon_{2}\right), \end{array}$$

respectively.

The expected minimum number of transmissions that will occur during the gathering is thus,


$$\begin{array}{l} I^{G}=I_{S1}^{G}+I_{V1}^{G}+I_{S2}^{G}+I_{V2}^{G}+I_{VV2}^{G}. \end{array}$$

#### Modeling vaccination intervention specific to gatherings

If public health interventions, including vaccination strategy, are applied at the gathering event to prevent the transmission, we assumed that only the fully vaccinated individuals are allowed to attend the gathering, which includes both vaccinated individuals and the individuals vaccinated but not effectively protected (infected, and in the prodromal state). Hence, the proportion of attendance in the qualified LRG and HRG on day *t* is


$${\bar{p}}_{L}(t)=\frac{p_{GL}N_{G}}{{\underline{V}}_{1}\left(t\right)+P_{V1}(t)},\;{\bar{p}}_{H}(t)=\frac{p_{GH}N_{G}}{{\bar{\bar{V}}}_{2}\left(t\right)+P_{V2}(t)},$$

where $P_{V1}=\frac{(1-\epsilon_{2})\underline{V_{1}}\left(t\right)}{S_{1}\left(t\right)+(1-\epsilon_{2})\underline{V_{1}}\left(t\right)}\left[P_{1}\left(t\right)+E_{1}(t)\right]$ and


$$P_{V2}=\frac{\left(1-\epsilon_{2}\right){\bar{\bar{V}}}_{2}\left(t\right)}{S_{2}\left(t\right)+\left(1-\epsilon_{1}\right){\bar{V}}_{2}\left(t\right)+\left(1-\epsilon_{2}\right){\bar{\bar{V}}}_{2}\left(t\right)}\left[P_{2}\left(t\right)+E_{2}\left(t\right)\right]$$

are the number of individuals in the prodromal state who are fully vaccinated but not effectively protected in the LRG and HRG, respectively.

Hence, the number of infectious attendees during the gathering on day t, which can only include the fully vaccinated individuals and those infected but in the prodromal stage, is


$$\begin{array}{l} \bar{N_{GI}}\left(t\right)=\bar{p_{L}}\frac{(1-\epsilon_{2}){\underline{V}}_{1}\left(t\right)}{S_{1}\left(t\right)+(1-\epsilon_{2}){\underline{V}}_{1}\left(t\right)}P_{1}\left(t\right)\\ \;+\bar{p_{H}}\frac{\left(1-\epsilon_{2}\right){\bar{\bar{V}}}_{2}\left(t\right)}{S_{2}\left(t\right)+\left(1-\epsilon_{1}\right){\bar{V}}_{2}\left(t\right)+\left(1-\epsilon_{2}\right){\bar{\bar{V}}}_{2}\left(t\right)}P_{2}(t). \end{array}$$

Since only fully vaccinated individuals are allowed to attend the gathering, the minimum probability of transmissions per attendee will become


$$\begin{array}{l} \bar{r_{G}}\left(t\right)=1-\left({\left(1-\frac{\bar{N_{GI}}\left(t\right)}{N_{G}-1}\;\beta \left(1-\epsilon_{2}\right)\right)}^{c_{G}}\right). \end{array}$$

Hence, the expected minimum number of transmissions that will occur during the gathering is


$$\bar{I_{V1}^{G}}=\bar{p_{L}}\left(t\right){\underline{V}}_{1}\left(t\right)\bar{r_{G}}\left(t\right),\bar{I_{VV2}^{G}}=\bar{p_{H}}\left(t\right){\bar{\bar{V}}}_{2}\bar{r_{G}}\left(t\right)$$

respectively.

### Scenario analysis

We conducted numerical simulations with the setting of the hypothetical metropolitan city, starting on 1 May 2022, and the model was run for 2 years (730 days). The vaccination started to be administered in HRG individuals on 10 June 2022 (28). The initial values, parameters with fixed values, and the range of some parameters used for simulations are presented in Tables 2–**4**. We investigated five different scenarios listed in **Table 6** by presenting the projection of daily new infections in LRG and HRG (per 100,000 individuals). In scenarios 1–3, the projection of mean and 95% confidence interval of daily new infections are obtained from 5,000 parameter sets sampling from the prior distribution (uniform) of parameters, by the Latin hypercube sampling (LHS) method (32, 33). We only present the mean of all the simulations in scenarios 2 and 3 for an intuitive interpretation of the results. We explored the proportion of vaccination coverage needed to prevent transmission under different assumptions in scenarios 4 and 5, where other parameters are fixed at the values presented in Tables 3, 4. The analyses were conducted using MATLAB (R2020a) (34).

**Table 2:** Variables used in the modeling of monkeypox transmission and their assumed initial values.

**Variables**	**Description**	**Initial value**	**Ref**.
*S*_*i*_(*t*)	Number of susceptible individuals in group i at day *t*	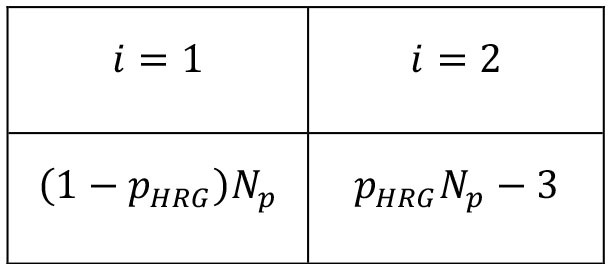	Assumed
*E*_*i*_(*t*)	Number of exposed individuals in group i at day *t*	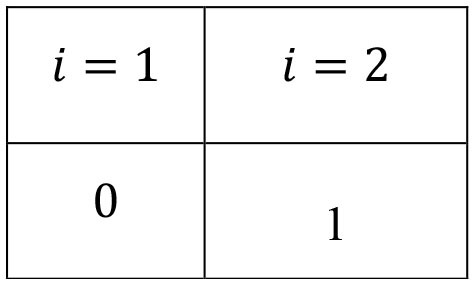	Assumed
*P*_*i*_(*t*)	Number of infectious individuals in the prodromal phase in group i at day *t*	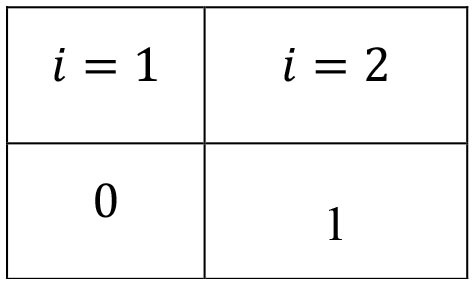	Assumed
*I*_*i*_(*t*)	Number of infectious individuals with rash symptoms in the acute phase in group i at day *t*	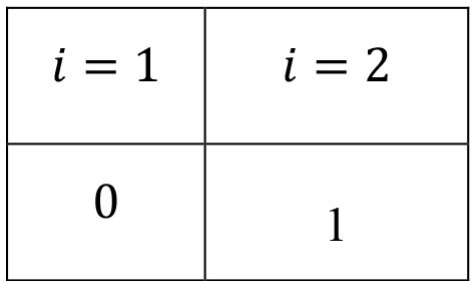	Assumed
*Q*(*t*)	Number of tested, confirmed and isolated symptomatic individuals at day t	0	Assumed
*R*(*t*)	Number of recovered individuals at day t	0	Assumed
*V*_1_(*t*)	Number of fully vaccinated individuals in LRG at day *t*	(1−*p*_*HRG*_) × 38.4% × *N*_*p*_	(25)
*V*_2_(*t*)	Number of partially vaccinated individuals in HRG at day *t*	0	Assumed
${\bar{\bar{V}}}_{2}\left(t\right)$	Number of fully vaccinated individuals in HRG at day *t*	*p*_*HRG*_ × 38.4% × *N*_*p*_	(25)
*Q*_*v*_(*t*)	Number of vaccinated and quarantined individuals who has been exposed to the confirmed cases at day t	0	Assumed
*Q*_*S*_*i*__(*t*)	Number of susceptible individuals in population i, i =1,2 who has been exposed to the confirmed cases at day t, and consequently vaccinated and isolated.	0	Assumed

**Table 3:** Parameters used in the modeling of monkeypox transmission (assumed).

**Parameter**	**Definition**	**Value (range)**	**Ref**.
**Demographic related**			
*p*_*HRG*_	Proportion of the population that is in the HRG	0.035	Assumed
*N*_*p*_	The total number of populations in the hypothetical city	5,000,000	Assumed
**Transmission related**
1/γ_*Q*_	Average number of days of recovery needed for isolated individuals, days	28	Assumed
1/γ_*v*_	Average number of days of recovery needed for exposed individuals vaccinated and quarantined, days	21	Assumed
ξ_1_	Scaling factor of infectiousness of infected in the prodromal phase compared to infections with rash symptoms	0.75	Assumed
ξ_2_	Scaling factor of susceptibility of LRG individuals compared to HRG individuals	0.03	Assumed
β	Probability of transmission per contact among HRG	(0.122, 0.245)	Assumed
*k*_1_	Proportion of contacts within the LRG in overall contacts	0.6	Assumed
*k*_2_	Scaling factor of contact rate among HRG compared to baseline contact	1.3	Assumed
**Vaccine related**			
ϵ_1_	The effectiveness of first dose of Imvamune against monkeypox infection	40% (40–60%)	Assumed
ϵ_2_	The effectiveness of second dose of Imvamune against monkeypox infection	80% (70–85%)	Assumed
**Public health control measures related**
1/λ	The average time to achieve the vaccination coverage in HRG, days	40 (30–90)	Assumed
*p*_*v*_	The vaccination coverage in HRG	0.6 (0.1–0.9)	Assumed
θ_1_	The vaccination proportion of individuals in LRG who are the close contact with the confirmed cases	0.35 (0.1–0.6)	Assumed
θ_2_	The vaccination proportion of individuals in HRG who are the close contact with the confirmed cases	0.6 (0.3–0.8)	Assumed
*r*_*LH*_	The daily transition rate of the low-risk susceptible individuals to the high-risk, 1/days	0.0005 (0.0001–0.001)	Assumed
*c*_*G*_	Number of effective contacts during the gathering, persons	30 (10–50)	Assumed
*p*_*t*1_	Proportion of symptomatic individuals in LRG who has been tested and isolated	0.3 (0.1–0.5)	Assumed
*p*_*t*2_	Proportion of symptomatic individuals in HRG who has been tested and isolated	0.5 (0.3–0.7)	Assumed
α	Proportion of individuals who comply with the isolation strategy	0.4 (0.3–0.6)	Assumed

**Table 4:** Parameters used in the modeling of monkeypox transmission (from literature).

**Parameter**	**Definition**	**Value (range)**	**Ref**.
**Demographic related**			
*p*_50+_	The proportion of individuals who were born before 1972 and has been fully vaccinated to the total populations	38.4%	(25)
**Transmission related**
τ	Average incubation period of MPX, days	13	(8)
δ	Average number of days from prodromal phase to acute phase, days	3	(8)
μ	Daily disease induced death rate, 1/days	3.6%/21	(7)
1/γ	Average number of days of recovery needed for infectious individuals with rash symptoms, days	21	(8)
*c*_0_	Baseline contact rate among the overall population, per day	10.8	(26)
**Vaccine related**			
1/ζ_1_	The time from the first dose administered to the immune response start, days	14	(11)
1/ζ_2_	The time from the immune response started after first dose to immune response peak after second dose, days	56	(11)
**Gathering event related**			
*T*_1_	The starting time of the gathering event	Jul. 20, 2022	(27)
*T*_2_	The end time of the gathering event	Aug. 1, 2022	(27)
**Public health control measures related**			
*T*_*v*_	The starting time of the vaccination strategy conducted	June 10, 2022	(28)
1/η	The average time of the close contact of confirmed cases from the exposed to be traced and then vaccinated, days	7 (1–14)	(11)
1/ρ	The average days from when infected individuals develop symptoms to seek medical help and then get tested, days	7 (1–13)	(29)

**Table 5:** Parameters defined in the modeling of monkeypox transmission.

**Parameter**	**Definition**	**Value (range)**	**Ref**.
*c*_*ij*_	Matrix of contacts among the groups	*c*_11_ = *c*_0_*k*_1_, *c*_21_ = *c*_12_,	
		*c*_12_ = *c*_0_(1−*k*_1_),	
		*c*_22_ = *c*_0_*k*_2_.	
**Gathering event related**		
*G*(*t*)	Indicator parameter if gathering event take place or not	-	Defined
*N*_*G*_	The number of attendees of the gathering event	-	Defined
*T*	The duration of gathering event, days	*T*_2_ − *T*_1_	Defined
*p*_*GH*_	Proportion of individuals attending the gathering event who is from HRG	1−*p*_*GL*_	Defined
*p*_*L*_	Proportion of LRG individuals attending the gathering event at day t	-	Defined
*p*_*H*_	Proportion of HRG individuals attending the gathering event at day t	-	Defined

### Sensitivity analysis

Sensitivity analyses were conducted to address the uncertainty of the parameters through the LHS and the partial rank correlation coefficient (PRCC) method (32). We generated 5,000 samples of parameters related to vaccination strategy, including the efficacy of the vaccine, the vaccination coverage in HRG, the days needed to achieve the vaccination coverage, and the vaccination proportion of close contacts of confirmed cases in HRG and LRG, and the parameters associated with gathering events, including the number of attendees of gathering, the effective contacts in the gathering, the proportion of attendees from LRG individuals. The ranges of parameters used in the sensitivity analysis are reported in Supplementary Table S1. We calculated the value of PRCC to investigate the relationship between the parameters and the model outputs of cumulative cases, which above 0.5 were considered to be significant.

## Results

### Impact of no public health measures implementation after gatherings

**Figure 2:**
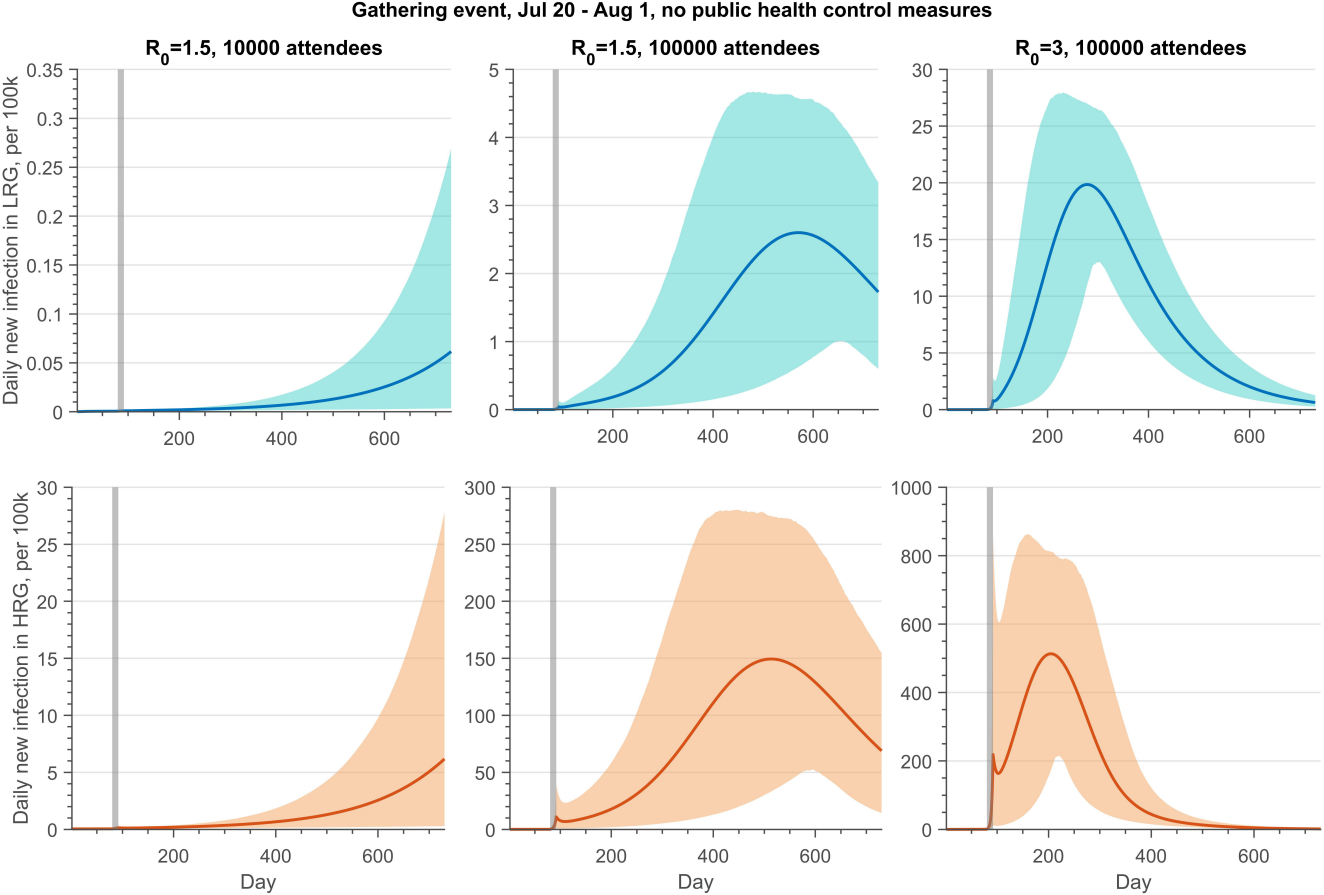
Projections of daily new infections (per 100 k) in the LRG and HRG without public health control measures if there is a gathering event from 20 July to 1 August 2022, with 10,000 or 100,000 daily attendees. The reproduction number of monkeypox transmission is 1.5 or 3. The gray shaded bar represents the gathering event occurring. The detailed setting can be found in Table 6, scenario 1.

We projected the daily new infections in LRG and HRG when the MGE occurred between 20 July and 1 August 2022 (Figure 2, Scenario 1 described in Table 6). We varied the number of participants and transmissibility levels of the monkeypox virus, under the assumption that public health control measures are not in place. A large outbreak of cases follows MGEs, with the number of cases increasing as the number of attendees and probability of transmission increase, raising the transmission. However, with the lowest number of participants and lower value of *R*_0_, the outbreak shows the beginning of an increasing trend 400 days after the gathering. For a gathering with 10,000 daily attendees, the risk of a monkeypox outbreak is low if the transmission probability per contact among the HRG individuals of the monkeypox virus is 12.2% (*R*_0_ = 1.5). On the other hand, we could observe a large outbreak if the number of participants increases to 100,000, with an average peak of daily new infections in HRG of around 150 per 100,000 people. If *R*_0_ = 3 and attendance increases to 100,000, the outbreak will immediately follow the gathering event, with a larger peak size of infections (500 per 100,000).

**Table 6:** Lists of settings of scenarios to projections of monkeypox infection.

**Scenarios**	**Settings**
Scenario 1	Gathering events and no public health (PH) control measures are conducted. • 10, 000 daily attendees, *R*_0_ = 1.5 (Baseline). • 10, 000 daily attendees, *R*_0_ = 3. • 100,000 daily attendees, *R*_0_ = 3.
Scenario 2	Gathering events (no interventions on the attendees) with 100,000 daily attendees, 10, 30 or 50 effective contacts in the gathering and *R*_0_ = 3. • With testing, isolation, and PEPCT strategy, but without PrEPH. • With testing, isolation, and PrEPH (90% HRG received 1 dose vaccine as of July 20), but without PEPCT strategy.
Scenario 3	Gathering events with 100,000 daily attendees (fully vaccinated), 10, 30 or 50 effective contacts in the gathering and *R*_0_ = 3. • With testing, isolation, and PrEPH (60% HRG received 1 dose vaccine as of Jul 20) and PEPCT strategy. • With testing, isolation, and PrEPH (90% HRG received 1 dose vaccine as of Jul 20), testing and isolation, but without PEPCT strategy.
Scenario 4	Gathering events (no interventions on the attendees) with 100,000 daily attendees, and *R*_0_ = 3. With testing, isolation, and without PEPCT strategy, and other settings.
Scenario 5	Gathering events (no interventions on the attendees) with 100,000 daily attendees, 50 effective contacts in the gathering and *R*_0_ = 3. With testing, isolation, vaccinating 30% close contact of confirmed cases in LRG, and other settings.
**Some general setting and descriptions**	
Gathering event	Starting from July 20 to August 1, 2022.
No PH control measures	No interventions on monkeypox transmission, like testing, isolation, tracing or vaccination.
*R*_0_ = 1.5 or 3	The probability of per contact among HRG is 12.2 % or 24.5%.
Testing, isolation	The testing and isolation strategy are implemented and the testing proportion of symptomatic infection in HRG and LRG is 50% and 30% respectively.
PEPCT strategy	Vaccinating the 60% close contact of confirmed cases in HRG and 30% close contact of confirmed cases in LRG.
Other settings	The time from individuals developing symptoms, to seeking medical help and then being tested and isolated is 7 days, and the time for tracing and vaccinating the close contacts of confirmed cases is 7 days, the daily transition rate from LRG to HRG during the gathering is 0.0005, and the proportion of individuals adhering to the isolation strategy is 40%, and the 10% of attendees is LRG individuals.

### Effects of PEPCT strategies

**Figure 3:**
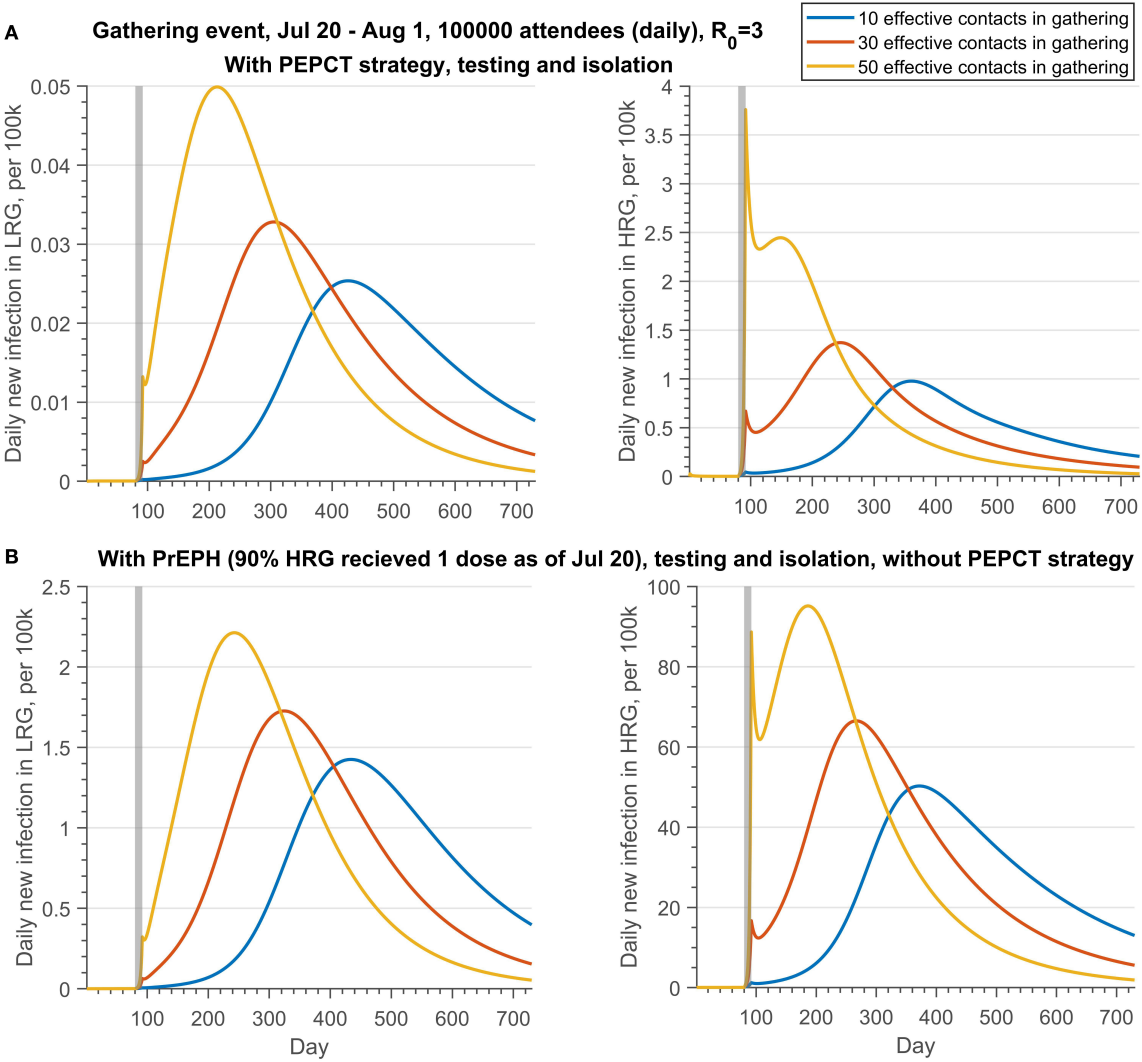
Projections of daily new infections (per 100 k) in the LRG and HRG with different public health control strategies under the different effective contacts in the gathering if there is a gathering event occurring from 20 July to 1 August 2022, with daily 100,000 attendees. **(A)** With PEPCT, testing and isolation, but without PrEPH; **(B)** with PrEPH, testing and isolation, but without PEPCT. Note that the reproduction number of monkeypox transmission is 3. The testing proportion of symptomatic infection in HRG and LRG is 50 and 30%, respectively. The PEPCT strategy represents that we vaccinated the 60% close contacts in HRG and 30% close contact in LRG. The gray shaded bar represents the gathering event occurring. The detailed setting can be found in Table 6, scenario 2.

**Figure 4:**
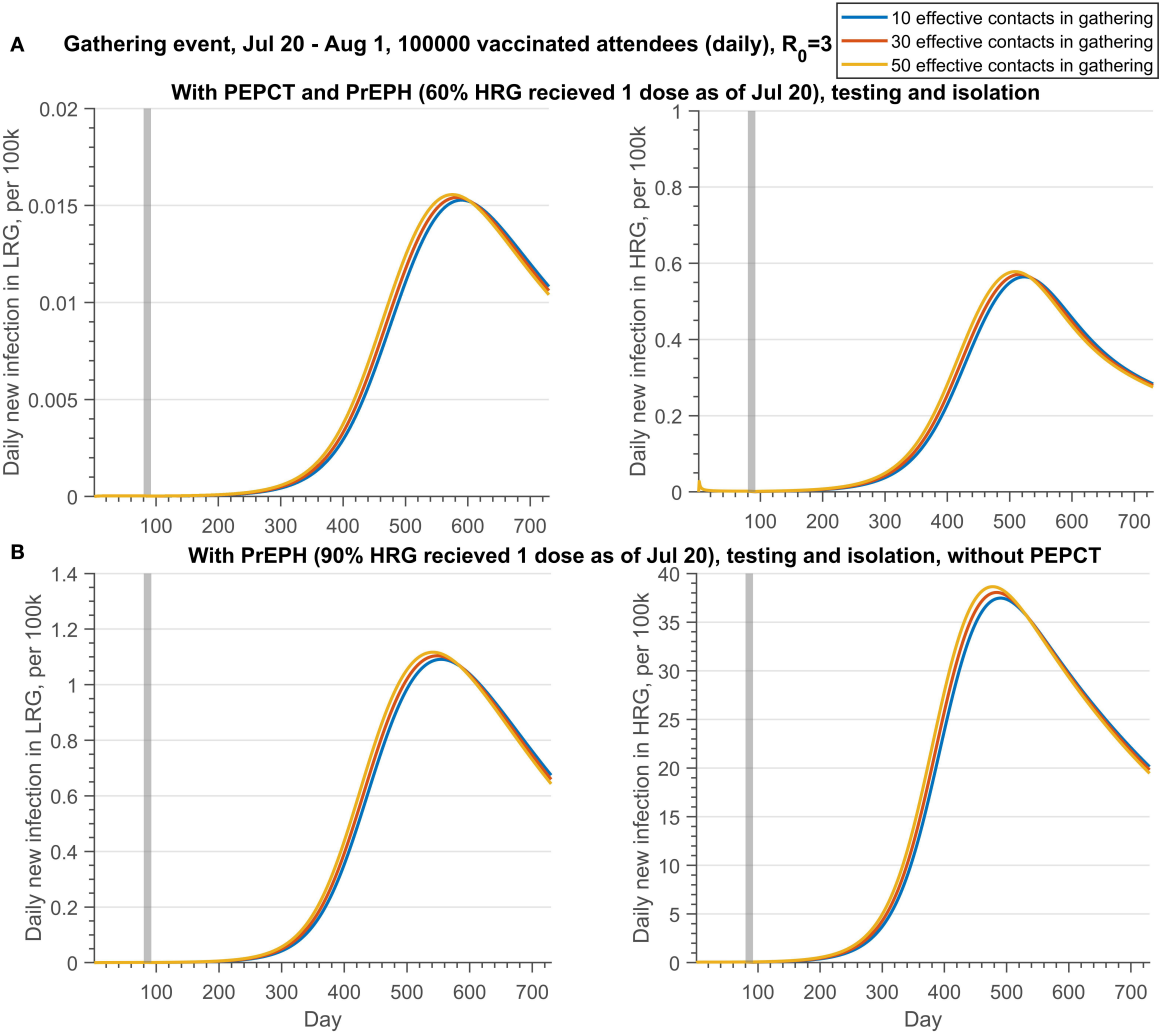
Projections of daily new infections (per 100 k) in the LRG and HRG with different public health control strategies under the different effective contacts in the gathering if there is a gathering event occurring from 20 July to 1 August 2022, with daily 100,000 attendees who has been fully vaccinated. **(A)** With PrEPH (60% HRG received 1 dose before 20 July) and PEPCT, testing and isolation; **(B)** with PrEPH (90% HRG received 1 dose before 20 July), testing and isolation, but without PEPCT. Note that the reproduction number of monkeypox transmission is 3. The testing proportion of symptomatic infection in HRG and LRG is 50 and 30%, respectively. The PEPCT strategy represents that we vaccinated the 60% close contacts in HRG and 30% close contact in LRG. The gray shaded bar represents the gathering event occurring. The detailed setting can be found in Table 6, scenario 3.

Figures 3, 4 (Scenario 2 and 3 described in Table 6) show the impact of PEPCT and PrEPH strategies under the possibility of a mass gathering with 100,000 attendees and relatively high transmission efficiency of the monkeypox virus (*R*_0_ = 3). Overall, if testing and isolation of symptomatic cases are in place, and contact tracing is effective, the PEPCT strategy is more beneficial to the control of monkeypox outbreaks, compared to the PrEPH strategy, and this is expected given the time needed to develop an immune response and the low vaccine coverage in the overall population in the simulations. With the implementation of the PEPCT strategy, tracing, vaccinating, and isolating 60 and 30% of close contacts of confirmed monkeypox cases in HRG and LRG, respectively, and maximum effective contacts in the gathering, the average peak of daily new infection in HRG was below 4 per 100,000 attendees (Figure 3, second panel). However, this number exceeds 90 per 100,000 if 90% of HRG individuals received at least 1 dose of vaccine before the gathering event started, but without PEPCT (Figure 3, fourth panel).

Similar results were obtained if all attendees of the gathering event are fully vaccinated (Figure 4). If the PEPCT strategy is implemented and the effective contacts during the gathering are 50, the mean peak of daily infection in HRG is below 1 per 100,000, although only 60% of individuals were administered 1 dose of vaccine before the gathering. Conversely, with only 10 effective contacts in the gathering, the mean peak of daily new infection in HRG exceeds 35 per 100, 000, if vaccinating 90% of HRG individuals with 1 dose before the gathering but without a PEPCT strategy. Regardless of whether there was a vaccination requirement for the attendance of gathering events, PEPCT strategies are critical to containing monkeypox transmission arising from gatherings.

Results for LRG follow the same trends of HRG, but with a smaller magnitude.

### Measures to prevent outbreaks after gatherings: Restricting effective contacts or vaccination

The peak size of the monkeypox outbreak is significantly associated with the number of effective contacts in the gathering if there are no restrictions on the gathering activities (Figure 3). Thus, the public health control measures aiming at constraining effective contacts during the gathering are essential to prevent the possible outbreak under this circumstance.

However, contact during the gathering would have a slight effect on the progression of the monkeypox transmission if only fully vaccinated individuals are allowed to attend the gathering (Figure 4). Moreover, whether there is a vaccination requirement for the attendees of gathering activities or not, public health control measures, such as PEPCT, are needed to prevent an outbreak resulting from MGEs.

### Identification of the best combination of vaccination coverage and gathering intervention

**Figure 5:**
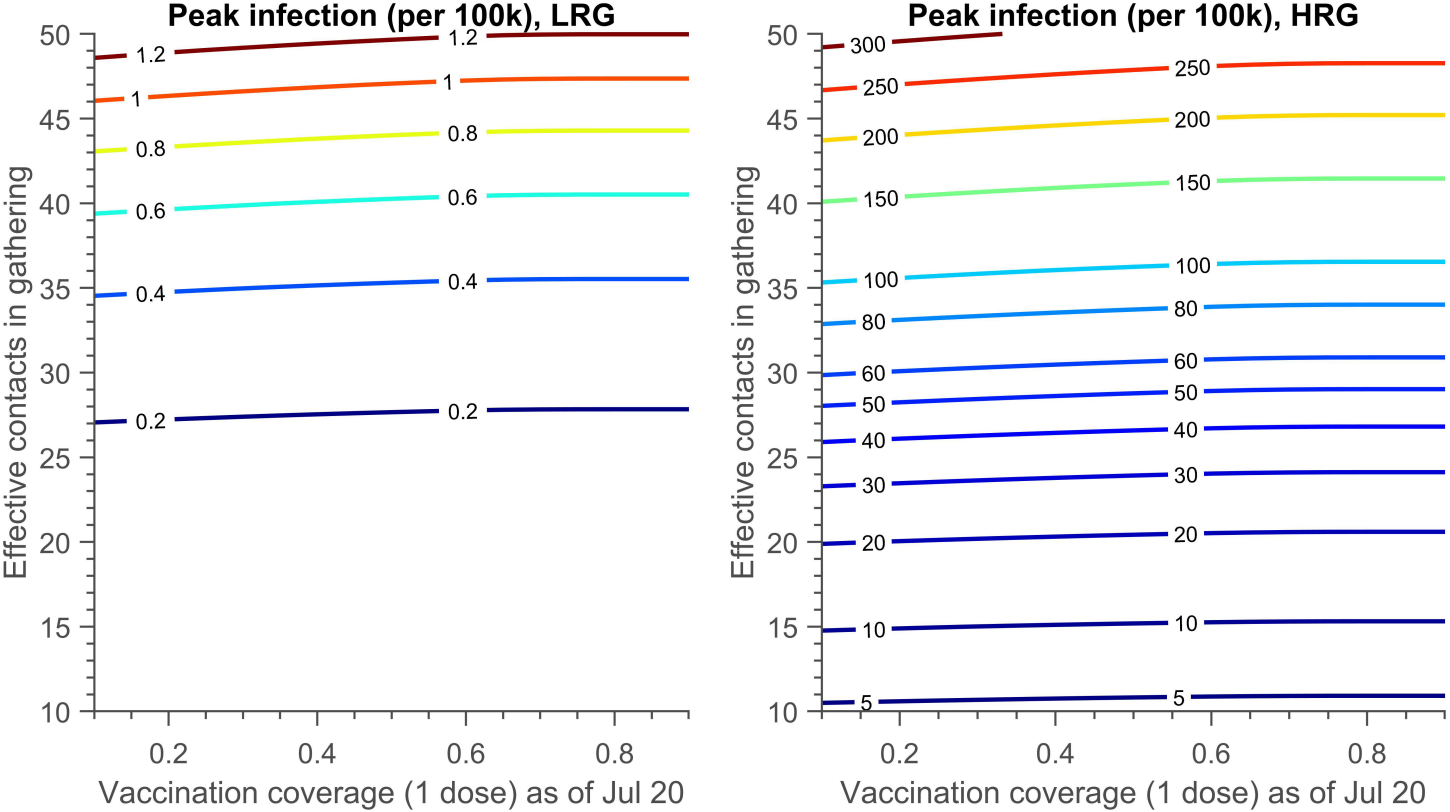
The contour plot of peak infection in LRG and HRG (per 100 k) with different effective contacts in the gathering and the vaccination coverage achieved (1 dose) on 20 July. There is no PEPCT strategy and the testing proportion of symptomatic infection in HRG and LRG is 50 and 30% respectively. The detailed setting can be found in Table 6, scenario 4.

Figure 5 shows a contour plot of peak infection in LRG and HRG under Scenario 4 (described in Table 6), when effective contacts and vaccine coverage (one dose by July 20) are varied. These results permit the determination of the vaccination coverage in HRG and constraints of contacts in gathering needed to prevent monkeypox transmission if the gathering event occurs. In this scenario, testing and isolation (30 and 50% of symptomatic infections in LRG and HRG) are included. As illustrated in Figure 5, public health interventions on constraining contacts during the gathering are more beneficial to contain the transmission, compared to the strategy of increasing vaccination coverage in HRG shortly before the event. The infection in the LRG could be kept below 1 per 100,000 if the effective contacts in the gathering are 46. However, the contacts should be < 15 to maintain a low prevalence (10 per 100,000) in HRG.

### Identification of the best combination of PEPCT and PrEPH strategy

**Figure 6:**
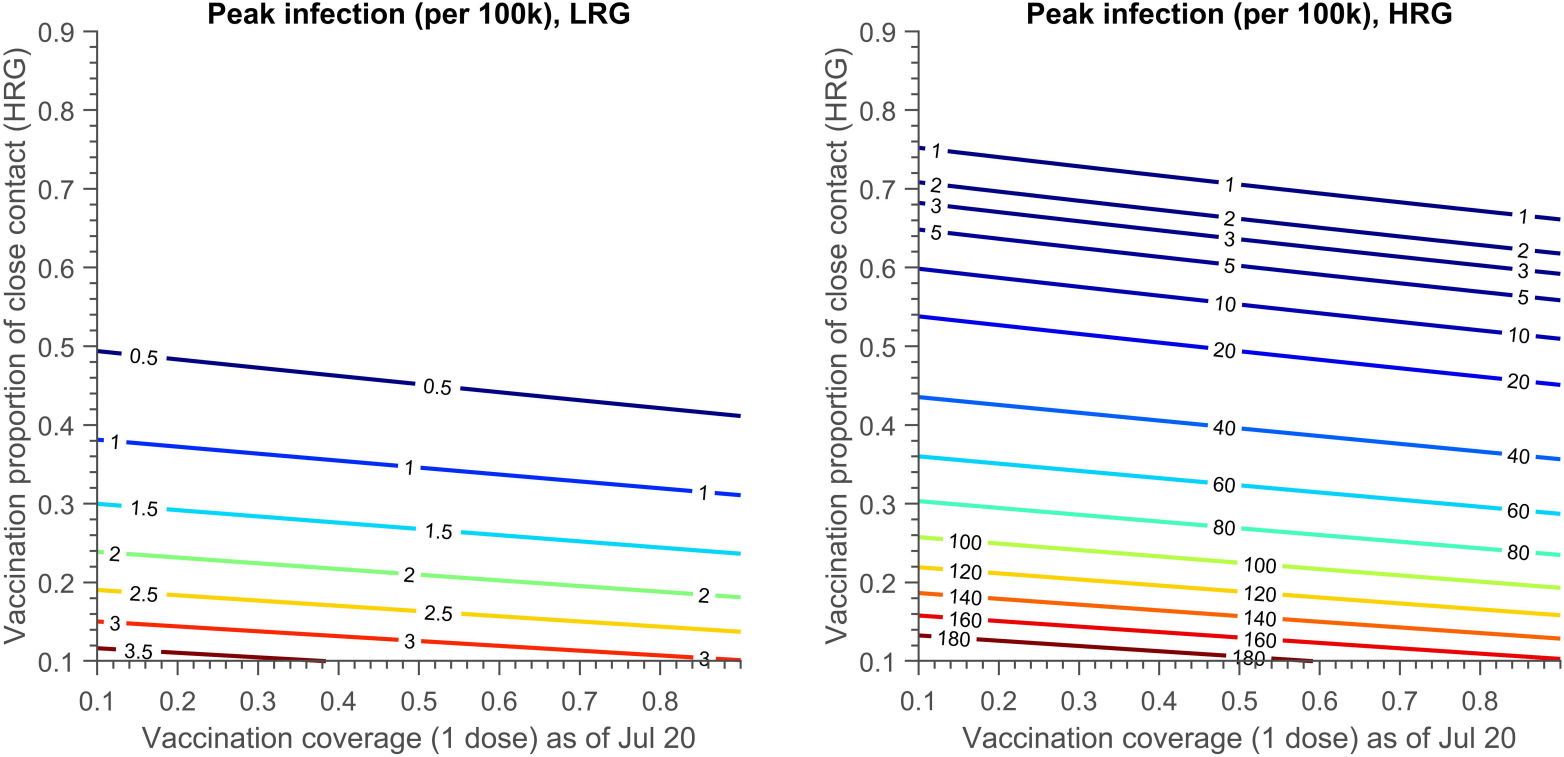
The contour plot of peak infection in LRG and HRG (per 100 k) with vaccination proportion of close contact in HRG and the vaccination coverage achieved (1 dose) on 20 July. The testing proportion of symptomatic infection in HRG and LRG is 50 and 30%, respectively. A total of 30% close contact of confirmed cases in LRG are traced, vaccinated and quarantined. The detailed setting can be found in Table 6, scenario 5.

The contour plot of peak infection in LRG and HRG with varying vaccination proportions of PEPCT and PrEPH is shown in Figure 6, if all the individuals were allowed to attend the mass gathering events (100,000 attendees), along with assumptions listed in Table 6 (Scenario 5). If vaccination coverage of 1 dose in HRG as of 20 July is 10%, to maintain the infection in LRG below 1 per 100,000, at least 38% of close contact of confirmed cases in HRG should be traced, vaccinated, and isolated. However, this proportion needs to increase to 75% to keep the infection in HRG below 1 per 100,000.

### Sensitivity analysis

**Figure 7:**
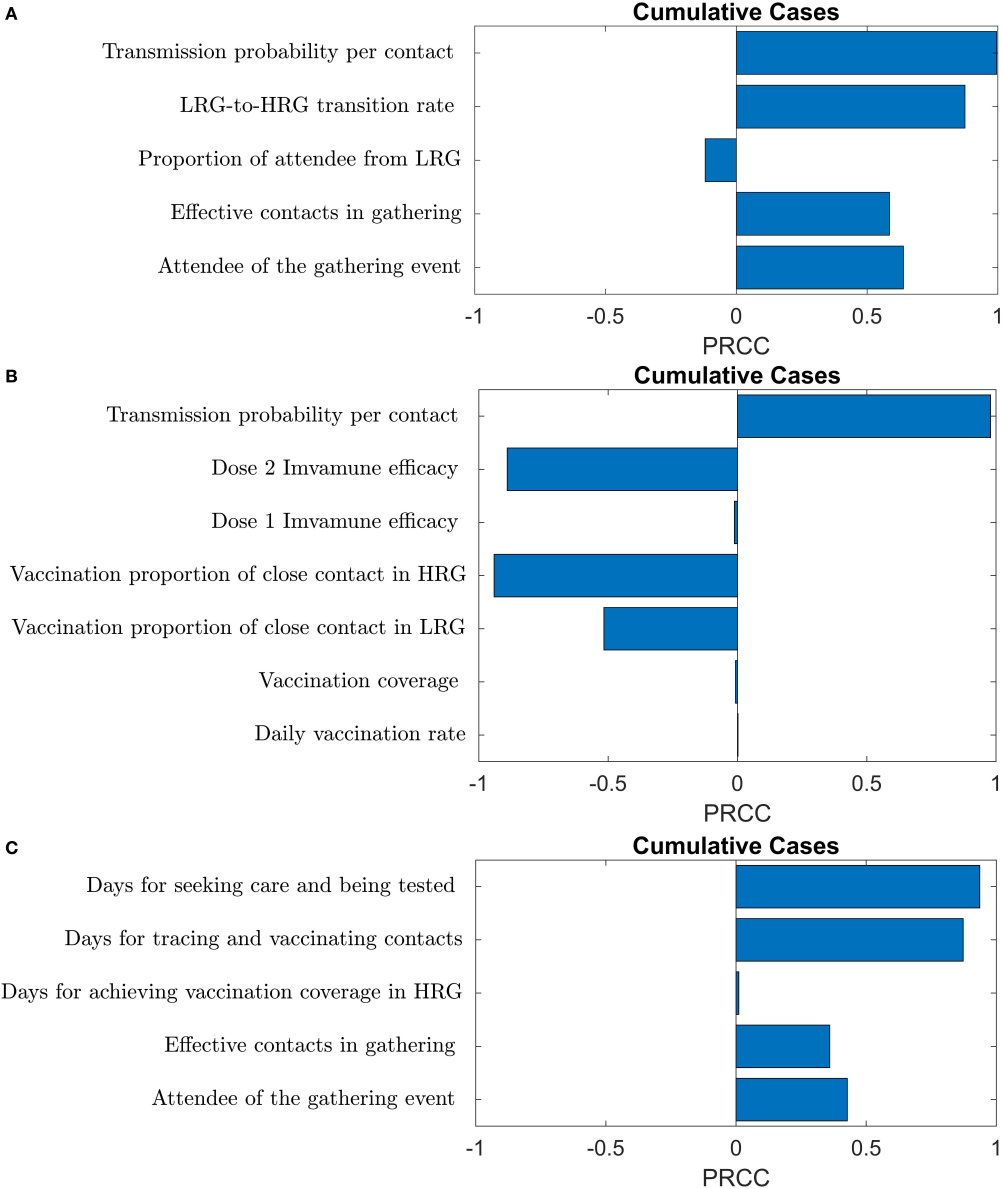
The PRCC plots of **(A)** gathering event-related **(B)** transmission and vaccine-related **(C)** public health control strategy-related parameters on cumulative cases.

The observed number of attendees, the number of effective contacts, and the transition rate from LRG to HRG during the gathering are significantly positively correlated with the cumulative cases (Figure 7A). On the other hand, the proportion of attendees from the LRG is negatively correlated to the cumulative cases, but not significant, which indicates a possibility of the dilution effect of the LRG attendees on the gathering events in consideration of the fact that they have a lower possibility to transmit the virus to the LRG community.

The sensitivity analysis also showed that the efficacy of the second dose and the vaccine coverage of close contact with confirmed cases in HRG and LRG present a negative correlation with cumulative cases (Figure 7B). Nevertheless, the first dose efficacy and its coverage in HRG, and the daily vaccination rate in HRG are not significant to the cumulative cases, due to the small proportion of HRG individuals in the total population (Figure 7C).

The sensitivity analysis confirmed that the PEPCT strategy and the interventions in the gathering activities are crucial to curb monkeypox spreading at gatherings. Moreover, the efforts of case detection, testing and isolation, and contact tracing, of symptomatic monkeypox cases affect the progression of the disease significantly (Figure 7C).

## Discussion

The classification of the monkeypox outbreak as a PHEIC by WHO underlines its global seriousness (3). In this study, we employed a mathematical modeling approach to explore how vaccinations and other public health measures can be implemented, in a hypothetical metropolitan city, to prevent the spread of monkeypox in human populations after mass gatherings events. Our results suggested that the risk of a monkeypox outbreak after gatherings is high, especially if the number of attendees was large and public health measures were not in place. However, effective public health measures can support the containment of monkeypox transmission at mass gatherings by a combination of constraints on effective contact at the gatherings, implementation of PrEPH, testing and isolation of symptomatic cases, and contact tracing and PEP (here studied as one process PEPCT). Vaccination requirements for participants in mass gathering events could play a crucial role in curbing the spreading of viruses. In addition, tracing, vaccinating, and isolating the close contacts who are exposed to cases was more beneficial to contain monkeypox transmission compared to a PrEP vaccination campaign in HRG individuals that begins shortly before the event.

Our novel model structure, with consideration of saturation of contacts at gatherings, allowed us to assess the monkeypox transmission risk on the occasion that gathering activities occur. Our results suggested that ring vaccination, along with efficient contact tracing and isolation, can be a powerful tool to halt the spread of the monkeypox virus linked to mass gathering events. Either limiting the gathering size and density, requiring the vaccination of attendees, or both may be essential for safe social gathering events. Additional measures were also required, such as a high level of effort to test and isolate confirmed cases and PEPCT, which required rigorous contact tracing and compliance with isolation policies.

In these simulations, the containment effect of PEPCT was determined by the vaccine efficacy, the proportion of contacts traced, the days needs to trace the exposures and vaccination, and the compliance with the quarantine after vaccination. Hence, PEPCT may not work as expected due to the uncertainty of efficacy and availability of the vaccine, as well as the difficulty in identifying the people who are most at risk from infection (35). Moreover, the significant containment effect of the PEPCT strategy mainly results from the transmission cut-off due to the contact tracing and isolation of the exposures. In reality, there is considerable difficulty in identifying contacts with cases, thus limiting the PEPCT strategy. Containing the spreading of monkeypox by ring vaccination protocol is also greatly dependent on the willingness of inoculation of the individual with exposure and compliance with the quarantine after vaccination.

In addition, the precise effectiveness of the smallpox vaccine against monkeypox infection in the human population remains unclear, although initial studies begin to suggest high effectiveness (36). The rollout of the vaccine campaign in HRG, or extending the eligibility to individuals with a moderate risk, requires the support of enough stockpiles of the vaccine. Given the limited vaccine capacity, the public health sector should prioritize vaccines for the communities at risk (37). However, targeting specific communities or groups of people may deepen stigma and hinder tracing, vaccination, and identification of cases (5, 35, 38). Also, only vaccinating the individuals with high risk was not enough to prevent the outbreak in a scenario with an increased transmission efficiency of the virus (possibly due to accelerated evolution) and there may be increased spillover from HRG to LRG (Supplementary Figure S1).

Although our modeling scenario simulations are conducted in a hypothetical metropolitan city with parameters from literature regarding Canadian cities, our model can be easily applied to any jurisdictions and areas where data are available, with the refinement of key parameters like the efficacy of vaccines to inform public health decision making. But the specific numbers required to halt the transmission need to be re-examined in a given region with local data. Besides, it is known that animals are a reservoir and part of the transmission to the human population, but this is not included in our work as we are focusing on the effect of vaccination and gathering events, and it can underestimate the transmission risk (23). We will include it in further study.

## Data availability statement

The original contributions presented in the study are included in the article/Supplementary material, further inquiries can be directed to the corresponding author/s.

## Author contributions

HZ, PY, YT, LY, EA, and NO: conceptualization. YT, LY, and PY: data curation. LY, PY, YT, and HZ: formal analysis. PY, YT, LY, EA, and HZ: methodology and writing—original draft. PY and YT: software, validation, and visualization. JB, JA, JH, JW, NO, HC, EA, PY, YT, LY, and HZ: writing—review and editing. HZ, JA, JB, JH, JW, and HC: funding acquisition. HZ: supervision. All authors contributed to the article and approved the submitted version.

## Funding

This research was supported by the Natural Sciences and Engineering Research Council of Canada OMNI-REUNI network for the Emerging Infectious Disease Modeling Initiative (NSERC EIDM) (JB, JA, JH, JW, HC, and HZ), Public Health Agency of Canada (HZ), and by the York Research Chair Program (HZ).

## Conflict of interest

The authors declare that the research was conducted in the absence of any commercial or financial relationships that could be construed as a potential conflict of interest.

## Publisher's note

All claims expressed in this article are solely those of the authors and do not necessarily represent those of their affiliated organizations, or those of the publisher, the editors and the reviewers. Any product that may be evaluated in this article, or claim that may be made by its manufacturer, is not guaranteed or endorsed by the publisher.
